# Microbiological and scanning electron microscopic evaluation of epidural catheters

**DOI:** 10.1136/rapm-2019-101180

**Published:** 2020-03-15

**Authors:** Ganapathy van Samkar, Payal P S Balraadjsing, Henning Hermanns, Irene V Hoogendijk, Markus W Hollmann, Sebastian A J Zaat, Markus F Stevens

**Affiliations:** 1 Department of Anesthesiology, AMC, Amsterdam, The Netherlands; 2 Department of Medical Microbiology, AMC, Amsterdam, The Netherlands

**Keywords:** neuraxial blocks: epidural, regional anesthesia, neuraxial blocks: continuous techniques

## Abstract

**Background:**

Epidural catheters are frequently colonized by gram-positive bacteria. Although the incidence of associated epidural infections is low, their consequences can be devastating. We investigated bacterial growth on epidural catheters by quantitative bacterial culture and scanning electron microscopy (SEM) in order to explore the patterns of epidural catheter colonization.

**Methods:**

28 patients undergoing major abdominal surgery with thoracic epidurals (treatment ≥72 hours) were studied. Before the removal of the catheter, the skin surrounding the insertion site was swabbed. The entire catheter was divided into extracorporeal, subcutaneous, and tip segments. Skin swabs and catheter segments were quantitatively cultured, bacterial species were identified, and SEM was performed on four selected catheters.

**Results:**

27 of 28 catheters were included. The percentages of positive cultures were: skin swab 29.6%, extracorporeal segments 11.1%, subcutaneous segments 14.8%, and tip segments 33.3%. One patient was diagnosed with a catheter-associated infection. *Staphylococcus epidermidis* was cultured from the skin and the catheter extracorporeal, subcutaneous, and tip segments. SEM of this catheter showed bacteria-like and intraluminal host cell-like structures. SEM of two other catheters showed intraluminal fibrin networks in their tip segments.

**Conclusions:**

We present the first SEM pictures of an epidural catheter with a bacterial infection. Bacterial growth developed from the skin to the tip of this catheter, indicating the skin as a primary source of infection. By SEM, catheters with low levels of bacterial growth demonstrated an intraluminal fibrous network which possibly plays a role in catheter obstruction.

## Introduction

The use of epidural catheters has a potential risk of infection. The documented incidence of epidural catheter infection is rather low but such infections can potentially be devastating. The incidence of epidural catheter-associated superficial infections ranges from 5% to 12% and that of associated deeper tissue infections, potentially causing permanent neurological damage, ranges from 1:1.000 to 1:100.000.^1–3^ Although not proven, bacterial colonization of epidural catheters may be a source of epidural infection.^4 5^ The colonization rate of epidural catheters is higher than that of actual infection, varying from 5% to 30%, with coagulase-negative staphylococci (CNS) being the most frequent pathogen.^4–9^ There are various proposed routes of epidural catheter colonization. Skin flora may spread along the catheter or its lumen, or become a source of contamination during needle or catheter insertion. Colonization can also occur via hematogenous spread from a distant source or via contaminated infusion fluid or delivery systems.^10^ The most common route of colonization is thought to be skin flora migrating along the epidural catheter.^9^ Skin disinfection is a standard procedure prior to epidural catheter insertion. However, bacteria residing in the deeper layers of the skin, including hair follicles, cannot be reached by disinfectants.^11^ Such resident bacteria recolonize the skin and epidural catheters when the protective skin barrier is breached by needle insertion.

The pattern of bacterial colonization along the epidural catheter has never been investigated in detail. In this observational study, we explored whether bacteria present *on* or *in* the skin are the primary source of colonization of the epidural catheter which progresses along the outer catheter surface towards the tip and from there potentially into its lumen. Therefore, we examined skin swabs, extracorporeal segments, subcutaneous segments, and tip segments of epidural catheters from patients receiving anesthesia and analgesia for bacterial growth.

## Methods

In this 6-month exploratory prospective study, 28 ASA 1–3 patients with thoracic epidural catheters (B.Braun Medical B.V. epidural anesthesia set 18G) in situ for at least 72 hours were included after approval by the ethics committee (W14_264#14170320) and after giving informed consent. Initially, catheters of 30 patients were collected, but 2 patients were excluded as they did not fulfill the study criteria (no thoracic epidurals, ≤12 hours in situ). We chose 72 hours as a cut-off point to have a higher chance of finding bacterial growth and subsequent infection, as most infections concerning epidural catheters start around the third day.^12 13^ Patients 14 and 9 were treated with epidural therapy for 69 and 70 hours, respectively, instead of the intended 72-hour treatment duration. As this marginal time difference does not significantly change the level of bacterial colonization, we have decided to include their data. All included patients underwent abdominal surgery, except patient 8, who underwent thoracic surgery. The catheters were placed according to a standardized local protocol based on the guideline by the Association of Anaesthetists of Great Britain & Ireland (AAGBI).^14^ The epidurals were inserted in the operating room under sterile conditions. The anesthesiologist prepared for a sterile procedure: hand disinfection, sterile gown, face mask, head covered with operative headgear and sterile gloves. The patient was positioned by an assistant who was also wearing a face mask and headgear. After two times disinfecting the skin with 0.5% chlorhexidine in 70% alcohol and waiting for it to dry, the sterile tray with the epidural set was unpacked, and the dorsal side of patient was draped with sterile plastic surgical draping. The epidural catheter was inserted at the level appropriate for the operative procedure (T4–T8) but not tunneled. Fixation was done using the StatLock Stabilization Device covered by a Tegaderm transparent surgical dressing and bacterial filter connected to the epidural catheter. Intravenous antibiotic prophylaxis (cefazolin and metronidazole) was administered after epidural catheter placement and prior to incision. Standard postoperative epidural analgesia was given by means of patient-controlled epidural analgesia with sterile pharmaceutically prepared bupivacaine 0.125% or bupivacaine 0.125% combined with 1 µg/mL sufentanil. Epidural therapy was ended by the acute pain service after at least 72 hours, if postoperative pain remained below 4 (Numeric Rating Scale) after discontinuation of epidural infusion. Catheters were removed according to the study protocol: first, a skin swab was taken surrounding the point of insertion. This was followed by skin disinfection with 0.5% chlorhexidine in 70% alcohol to reduce the incidence of contamination of skin flora during withdrawal and subsequent removal of the catheter. Directly after catheter removal, the catheter was cut with sterile scissors into two segments: the proximal segment (extracorporeal and subcutaneous) and the distal segment (tip). The segments and skin swabs were transported to the microbiology research laboratory in sterile tubes.

**Figure 1:**
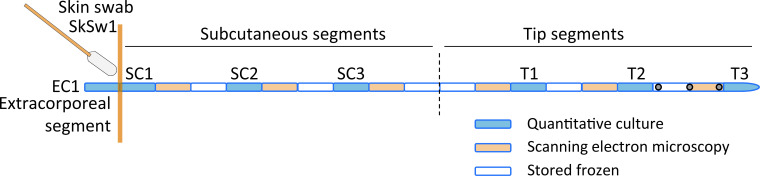
Schematic overview of epidural catheter segments and skin swab used for quantitative bacterial culture (blue segments) or scanning electron microscopy (orange segments). EC1 is the extracorporeal segment; SkSw1 is skin swab; SC1, SC2, SC3 are subcutaneous segments, from superficial (SC1) to deep (SC3); T1, T2, T3 are tip segments (T3 is the end of the epidural catheter).

Microbiological methods: The subcutaneous and tip catheter segments were cut in 0.5 cm segments under sterile conditions to assess a possible gradient of bacterial growth along the catheter (figure 1). The catheter segments were labeled as follows: EC1 for the extracorporeal segment (outside the patient), SC1, SC2, SC3 for the subcutaneous segments and T1, T2, T3 for the tip segments (figure 1). The skin swab was labeled as SkSw1 (figure 1). The segments were used for either quantitative bacterial culture, scanning electron microscopy (SEM), or were stored frozen (−80°C) for later evaluation (figure 1). Bacteria of the catheter segments were retrieved by the sonication method as previously described, with minor adaptations.^15^ In brief, catheter segments were sonicated for 30 s in 500 µL sterile 0.9% NaCl at 35 kHz in a sonicator water bath (Elma, Transsonic 460) followed by vortexing for 10 s. Sixty-microliter aliquots of the sonicate fluid (1:8.3 of total sonicate fluid) were plated on blood agar plates in duplicate and incubated either aerobically or anaerobically at 37°C for 48 up to 96 hours. In addition, 10-fold dilution series of the sonicate fluids were made and incubated under the same circumstances as described earlier to allow precise enumeration in case of bacterial growth above the countable range. If bacterial growth was observed, then colonies were counted and distinguished based on colony morphology. Bacterial growth was quantified in colony-forming units (CFU) per catheter segment based on the numbers of CFU recovered and the respective dilution. Bacterial growth on the skin is expressed in CFU per swab. We defined a bacterial culture as positive if we found ≥1 CFU on the agar plates. The lower detection limit of bacterial quantification was <8.3 CFU and the upper detection limit was ≥4165 CFU. The species of retrieved bacteria were identified using Matrix-Assisted Laser Desorption/Ionization Time-of-Flight Mass Spectrometry (Microflex LT, Bruker Daltonic).^16^ Finally, we performed SEM on four selected catheters: a control catheter (not inserted in a patient), a non-colonized catheter (patient 14), a catheter with low levels of bacterial growth (patient 29), and a catheter of a patient with clinical infection (patient 5). Catheter segments for SEM were fixated in 4% formaldehyde. To ensure fixation of the lumen of the catheter segments, they were placed under vacuum for 30 s. Fixated catheter segments were prepared for SEM according to standardized protocols. Catheter segments were imaged using a Zeiss Sigma-300 FE scanning electron microscope.

## Results

Of the catheters collected from the 28 patients included in the study, only 27 catheters were investigated. One catheter was excluded from analysis due to non-adherence to the protocol. One patient, patient 5, had signs of local infection (redness of the skin, tenderness, and pus around the insertion site). The catheter tip segment of this patient was cultured by the microbiology diagnostic laboratory using the standardized roll plate method^17^ instead of the sonication method used in the microbiology research laboratory.

Of the 27 cultured catheters, 10 (37%) catheters had some level of bacterial growth on the subcutaneous and/or tip segment. The majority of the patients had no bacterial growth (0 CFU) on any segment of the catheter or on the skin (table 1). The number of patients (4) with subcutaneous segments positive for bacterial growth (≥1 CFU) was lower than the number of patients (9) showing positive bacterial growth on the tip of the catheters (table 1).

**Table 1:** Patients with bacterial growth on epidural catheters and skin

	Extracorporeal segment	Skin swab	Subcutaneous segment	Tip segment
Patients, n (%)				
0 CFU	24 (88.9)	19 (70.4)	23 (85.2)	18 (66.7)
≥1 CFU	3 (11.1)	8 (29.6)	4 (14.8)	9 (33.3)

Positive bacterial growth is indicated by ≥1 CFU, and no bacterial growth as 0 CFU, n=27.

CFU, colony-forming units.

All positive cultures of the subcutaneous and tip segment exhibited low numbers of bacteria, no distinct bacterial growth pattern and corresponding patients had no signs of infection, with the exception of patient 5 (table 2). Moreover, patients with positive cultures of the subcutaneous or tip segment did not always have positive cultures of the extracorporeal segment or the skin swab (table 2), indicating no direct correlation between skin colonization and catheter colonization in these patients. In patient 5, a clinically relevant skin infection was diagnosed 96 hours after insertion of the epidural catheter. The catheter of this patient was removed immediately, and the patient was monitored closely for 48 hours. In contrast to the other catheters, which were cultured in the experimental laboratory, this epidural catheter was cultured in the clinical laboratory because of this clinically relevant infection. Clinical treatment of patient 5 consisted of 2 days of “watchful waiting” with no antibiotic therapy, as there were no signs of aggravating local or systemic infection. During this period, the patient developed no further signs of systemic or neuraxial infection. The bacterial growth levels decreased from skin to tip and culture of skin swab and epidural catheter revealed a monoculture of *S. epidermidis*.

**Table 2:** Pattern of bacterial growth on epidural catheters

Patient no.	Number of CFU cultured from								Duration of epidural, hours
	Extracorporeal segment	Skin swab	Subcutaneous segment			Tip segment			
	EC1	SkSw1	SC1	SC2	SC3	T1	T2	T3	
2	316.5	0	0	0	0	0	0	0	173
8	0	16.66	0	0	0	0	0	0	76.5
11	0	≥4165	0	0	0	0	0	0	73.5
12	0	3332	0	0	0	0	0	0	101
14	1532.7	0	0	0	0	0	0	0	69
18	0	≥4165	0	0	0	0	0	0	74
17	0	0	<8.3	0	0	0	0	0	99
1	0	≥4165	0	0	<8.3	16.7	0	0	77
13	0	≥4165	0	0	0	25.0	0	0	80
27	0	0	0	0	0	0	<8.3	0	73
29	0	≥4165	0	<8.3	0	0	0	<8.3	72
7	0	0	0	0	0	0	0	<8.3	76
9	0	0	0	0	0	0	0	<8.3	70
4	0	0	0	0	0	0	41.7	0	96
3	0	0	0	0	0	0	0	25.0	73
5*	≥4165	≥4165	1774.3	158.3	91.6	Diagnosed as positive and evaluated in clinical lab			96

Values indicate numbers of CFU or in situ duration (hours). No growth is indicated as 0 CFU. Lower detection limit 8.3 CFU. Upper detection limit 4165 CFU. The average duration of epidural therapy in non-colonized patients was 95 hours (range: 72–171 hours) (data not shown).

Many diagnostic microbiology laboratories define at least 100 CFU in quantitativestudies as a threshold indicating colonization.

*Patient with superficial infection.

CFU, colony-forming units; EC1, extracorporeal catheter segment; SC1, subcutaneous catheter segment 1 (superficial); SC2, subcutaneous catheter segment 2; SC3, subcutaneous catheter segment 3 (deep); SkSw1, skin swab; T1, catheter tip segment 1; T2, catheter tip segment 2; T3, catheter tip segment 3 (utmost tip in epidural space).

The bacterial species isolated from the epidural catheters are shown in table 3. The most frequently isolated bacteria were CNS and *Micrococcus luteus*, both part of the normal skin flora. The bacterial population present on the subcutaneous and tip segment mostly consisted of a monoculture whereas the population retrieved from the extracorporeal segment and the skin swab mostly consisted of mixed bacterial species.

**Table 3:** Bacterial species cultured from epidural catheters and skin swabs

	Extracorporeal segment	Skin swab	Subcutaneous segment	Tip segment
Patients positive for bacterial growth, n				
CNS*	2	4	1	1
*Micrococcus luteus*	0	0	1	5
*Propionibacterium acnes*	0	3	0	0
*Streptococcus* spp†	1	0	0	1
*Bacillus* spp‡	0	1	1	1
*Kocuria rhizophila*	0	0	0	2
*Neisseria* spp§	1	0	0	0
*Rhodotorula mucilaginosa*	1	0	0	0
*Actinomyces oris*	1	0	0	0
Unknown	1	3	1	0

*CNS include *S. epidermidis, S. saccharolyticus, S. capitis, S. warneri*.

†Streptococcus spp: *S. salivarius, S. mitis*.

‡Bacillus spp: *B. simplex, B. horneckiae, B. licheniformis*.

§Neisseria spp: *N. perflava*, *N. flavescens*.

CNS, coagulase-negative staphylococci.

**Figure 2:**
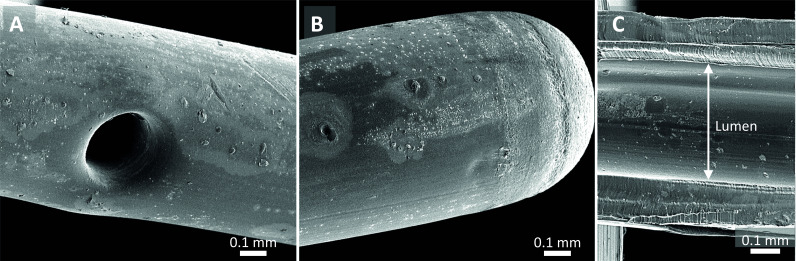
Scanning electron microscope images of the sterile epidural catheter, not inserted in the patient. (A) Outer surface of the catheter with a side hole. (B) Outer surface of the tip. (C) Cross-section of the catheter showing the catheter wall and lumen. White bar indicates scale.

**Figure 3:**
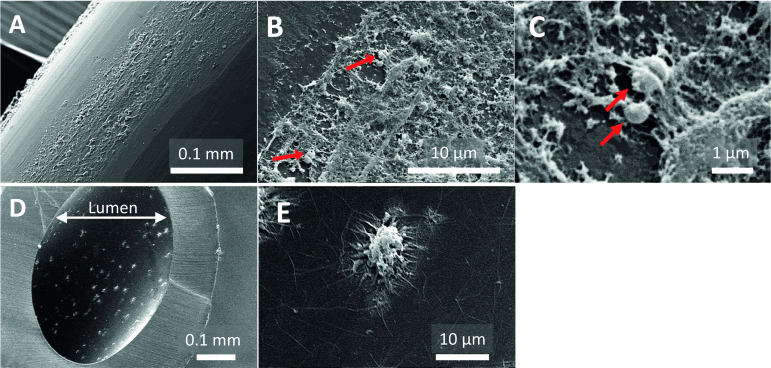
Scanning electron microscope images of subcutaneous segments of infected epidural catheter (patient 5). Outer surface of the catheter with (A) low, (B) medium, or (C) high magnification. The surface of the catheter is partially covered with biological deposits with staphylococci-like spheres, indicated by red arrows. Side view of the catheter lumen with adherent cell-like structures in (D) low and (E) medium magnification. Pseudopodia are emerging from the cell-like structures. White bar indicates scale.

**Figure 4:**
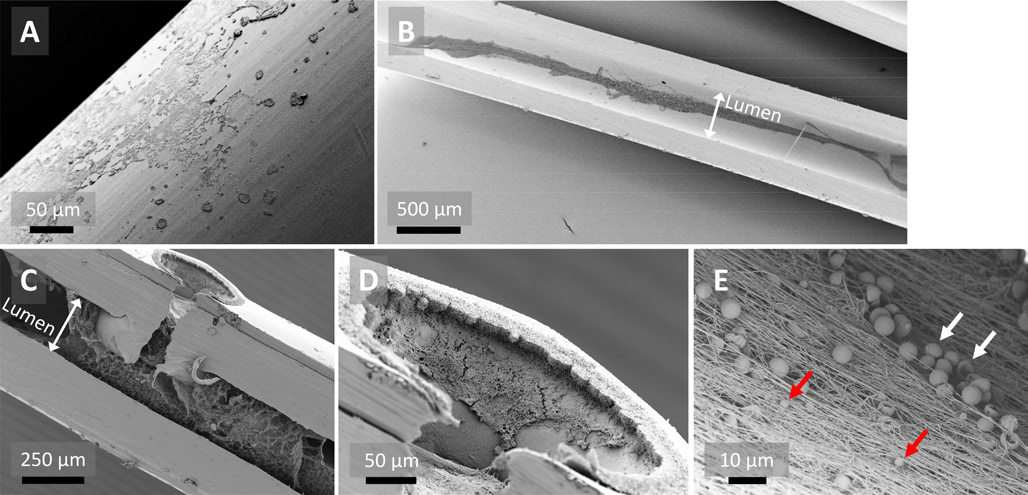
Scanning electron microscope images of tip segments of the epidural catheter with low levels of bacterial growth (patient 29). (A) Outer surface of the catheter with biological deposits. (B) Cross-section of the catheter segment showing intraluminal fibrin-like fibers stretching in the length of the catheter. (C) Cross-section of the catheter with a side hole showing the intraluminal fibrous network and clot. Fibers seem to progress from the side hole to the intraluminal space. (D) Higher magnification of the side hole, showing an organized layer of cell-like structures lining the border and interior of the side hole. (E) Higher magnification of fibrin-like fibers with erythrocytes and blebs embedded in the fibrous network, indicated by white and red arrows, respectively. Black bars indicate scale.

SEM examination of sterile epidural catheters revealed smooth areas but also irregularities on the outer surface and lumen of the catheter (figure 2). SEM of the infected catheter showed biological deposits on the outer surface with spherical structures resembling staphylococci (figure 3A–C). In the lumen of this infected catheter adherent host cell-like structures were observed (figure 3D). The extensions emerging from these structures resemble the pseudopodia of immune cells (eg, macrophages) (figure 3E). Analysis of the catheters with low levels of bacterial growth revealed biological deposits on the outer surface of the catheter (figure 4A). The lumen of the tip segments of this catheter revealed a network of fibrin-like fibers with erythrocytes and blebs (figure 4B–E). Interestingly, this was not the case in the corresponding subcutaneous or extracorporeal segments. Similar intraluminal structures were observed in a non-colonized catheter (data not shown).

## Discussion

Bacterial colonization of the skin is often suggested as a potential source of infection of epidural catheters. In this exploratory study, we show a pattern of colonization decreasing from the skin to catheter tip in one patient with a clinically relevant infection. SEM of this catheter revealed biological deposits with staphylococcal-like structures and intraluminal immune-cell-like structures. Catheters with low levels of bacterial growth had no distinct bacterial growth patterns. Interestingly, SEM revealed a dense intraluminal fibrous network in these non-infected catheters.

A third (33%) of the patients had some level of growth on the epidural catheter tip segments, which corresponds to data from the literature.^7^ In contrast to the patient with clinical infection, bacterial growth on the skin of patients without clinical infection was not a predictor for bacterial growth on the catheter tip. The low numbers of bacteria cultured from the tip segments may have been contamination occurring during catheter removal or processing. The catheters with low levels of bacterial colonization, along with significant skin colonization, showed little consistency of bacterial species between that on the skin and that affecting the catheter, indicating bacteria from distant sources. Further studies are needed to evaluate whether high skin colonization may be a predictor for catheter colonization in case of longer catheter duration.

In the absence of a standardized definition, relevant bacterial growth on epidural catheters tips, also designated as bacterial colonization, is often defined according to the criteria used for central venous catheter (CVC) tips. Depending on the culture method, reference cut-offs of ≥15 CFU (semiquantitative) or ≥100 CFU (quantitative) are used to define bacterial colonization and these are associated with clinical infection in CVC.^17 18^ For epidural catheters, this relationship between bacterial colonization and infection is uncertain when using these cut-offs.^4 7 8^ We defined a bacterial culture as positive if we found ≥1 CFU on the catheter segment or skin. If we would apply the reference cut-off to our data, then only the case of infection would reach this cut-off, and all patients without infection symptoms would not. This indicates that the cut-off used for relevant bacterial growth on CVC tips may also give a good estimation of relevant bacterial growth on epidural catheter tips, but this should be investigated in a more extensive study.

Biological deposits on the surface of catheters are a common phenomenon with CVCs. On blood contact, the catheter surface is coated with host-derived proteins (eg, fibrin) to which bacteria can attach and form a biofilm.^19^ A comparable process may occur with epidural catheters when blood ends up on the catheter tip during catheter insertion. Immunohistochemistry could reveal whether the biological deposits observed on the catheter surfaces are host-derived or bacteria-biofilm-derived. Interestingly, the observed intraluminal fibrin-like fibers seemed to enter from the side holes into the lumen and became less in density as they progressed to the subcutaneous catheter segments. This phenomenon of an intraluminal fibrous network (clot) in epidural catheters has never been shown before and might be one cause of catheter obstruction.

The main limitation of our study is the small number of patients and catheters investigated. However, this was an exploratory study to investigate and image the pattern of bacterial colonization on the full length of epidural catheters rather than only investigating catheter tips. The inspected catheters displayed some interesting new findings, such as suspicion of biofilm formation, host cell invasion, and fibrin clot formation inside the catheter tip which have never been demonstrated before.

In conclusion, our data indicate that the level of bacterial colonization on epidural catheters is low and only in the case of clinical infection the skin seems the primary source of bacterial colonization. Our data also suggest that an intraluminal fibrous network develops from the side holes into the lumen and possibly plays a role in catheter obstruction.

## References

[1] Darchy B, Forceville X, Bavoux E, et al. Clinical and bacteriologic survey of epidural analgesia in patients in the intensive care unit. Anesthesiology 1996;85:988–98. 10.1097/00000542-199611000-00005 8916814

[2] Grewal S, Hocking G, Wildsmith JAW. Epidural abscesses. Br J Anaesth 2006;96:292–302. 10.1093/bja/ael006 16431882

[3] Stratton A, Gustafson K, Thomas K, et al. Incidence and risk factors for failed medical management of spinal epidural abscess: a systematic review and meta-analysis. J Neurosurg Spine 2017;26:81–9. 10.3171/2016.6.SPINE151249 27636865

[4] Harde M, Bhadade R, Iyer H, et al. A comparative study of epidural catheter colonization and infection in intensive care unit and wards in a tertiary care public hospital. Indian J Crit Care Med 2016;20:109–13. 10.4103/0972-5229.175943 27076712 PMC4810923

[5] Kostopanagiotou G, Kyroudi S, Panidis D, et al. Epidural catheter colonization is not associated with infection. Surg Infect 2002;3:359–65. 10.1089/109629602762539571 12697082

[6] Morin AM, Kerwat KM, Klotz M, et al. Risk factors for bacterial catheter colonization in regional anaesthesia. BMC Anesthesiol 2005;5:1. 10.1186/1471-2253-5-1 15774007 PMC1079795

[7] Simpson RS, Macintyre PE, Shaw D, et al. Epidural catheter tip cultures: results of a 4-year audit and implications for clinical practice. Reg Anesth Pain Med 2000;25:360–7. 10.1053/rapm.2000.5672 10925931

[8] Steffen P, Seeling W, Essig A, et al. Bacterial contamination of epidural catheters: microbiological examination of 502 epidural catheters used for postoperative analgesia. J Clin Anesth 2004;16:92–7. 10.1016/j.jclinane.2003.05.007 15110369

[9] Yuan H-B, Zuo Z, Yu K-W, et al. Bacterial colonization of epidural catheters used for short-term postoperative analgesia: microbiological examination and risk factor analysis. Anesthesiology 2008;108:130–7. 10.1097/01.anes.0000296066.79547.f3 18156891

[10] Dawson S. Epidural catheter infections. J Hosp Infect 2001;47:3–8. 10.1053/jhin.2000.0872 11161894

[11] Karpanen TJ, Worthington T, Conway BR, et al. Penetration of chlorhexidine into human skin. Antimicrob Agents Chemother 2008;52:3633–6. 10.1128/AAC.00637-08 18676882 PMC2565868

[12] Moen V, Dahlgren N, Irestedt L. Severe neurological complications after central neuraxial blockades in Sweden 1990-1999. Anesthesiology 2004;101:950–9. 10.1097/00000542-200410000-00021 15448529

[13] Wang LP, Hauerberg J, Schmidt JF. Incidence of spinal epidural abscess after epidural analgesia: a national 1-year survey. Anesthesiology 1999;91:1928–36. 10.1097/00000542-199912000-00046 10598636

[14] AssociationOfAnaesthetistsOfGreatBritainAndIreland. Best practice in the management of epidural analgesia in the hospital setting., 2010. Available: https://wwwaagbiorg/sites/default/files/epidural_analgesia_2011pdf

[15] Raad II, Sabbagh MF, Rand KH, et al. Quantitative tip culture methods and the diagnosis of central venous catheter-related infections. Diagn Microbiol Infect Dis 1992;15:13–20. 10.1016/0732-8893(92)90052-U 1730183

[16] Seng P, Rolain J-M, Fournier PE, et al. MALDI-TOF-mass spectrometry applications in clinical microbiology. Future Microbiol 2010;5:1733–54. 10.2217/fmb.10.127 21133692

[17] Maki DG, Weise CE, Sarafin HW. A semiquantitative culture method for identifying intravenous-catheter-related infection. N Engl J Med 1977;296:1305–9. 10.1056/NEJM197706092962301 323710

[18] Bouza E, Alvarado N, Alcalá L, et al. A prospective, randomized, and comparative study of 3 different methods for the diagnosis of intravascular catheter colonization. Clin Infect Dis 2005;40:1096–100. 10.1086/428576 15791507

[19] Waldvogel F. Infections associated with indwelling medical devices. Third ed.. Washington DC: ASM Press, 2000.

